# An Ultrathin Tunable Metamaterial Absorber for Lower Microwave Band Based on Magnetic Nanomaterial

**DOI:** 10.3390/nano12132135

**Published:** 2022-06-21

**Authors:** Jing Ning, Ke Chen, Wenbo Zhao, Junming Zhao, Tian Jiang, Yijun Feng

**Affiliations:** School of Electronic Science and Engineering, Nanjing University, Nanjing 210093, China; dg20230031@smail.nju.edu.cn (J.N.); wenbozhao@smail.nju.edu.cn (W.Z.); jmzhao@nju.edu.cn (J.Z.); jt@nju.edu.cn (T.J.)

**Keywords:** metamaterial absorber, ultrathin, tunable absorption, magnetic nanomaterial

## Abstract

At frequencies below 1 GHz, conventional microwave absorbers are limited by their large thickness or narrow absorption bandwidth; therefore, new techniques for efficient absorption for the lower microwave band are highly demanded. Here, we propose and fabricate an ultrathin tunable metamaterial absorber combining magnetic nanomaterials and metamaterial resonant structures for use in the lower microwave band (P band). The proposed absorber utilizes electrically controlled varactors to enable frequency tunability and magnetic nanomaterials as dielectric slabs for thickness reduction and bandwidth expansion at low frequencies. By adjusting the bias voltages of varactors, the resonant behavior of the absorbing structure can be dynamically tuned that covers a continuously tunable absorbing band from 0.41 to 1.02 GHz (85.3% in fractional bandwidth) with at least 10 dB reflection reduction. The total thickness of this absorber is 5 mm, which is only about 1/146 the wavelength of the lowest frequency. The agreement between the simulated and measured results validates the proposed design, and the structure has good angular stability that may be used as complex targets for low-RCS applications.

## 1. Introduction

Microwave-absorbing materials are continuously attracting researchers’ interest due to their potential applications in communication and defense fields. During the past few decades, various types of absorbing materials have been proposed, such as carbon-based absorbing materials [1], magnetic absorbers [2,3], perfect absorbers based on metamaterials [4,5], Salisbury absorbers [6,7], Jaumann absorbers [8], and circuit analog absorbers [9]. However, the effective working frequencies of these absorbers are typically above 1 GHz, because the absorption of long electromagnetic (EM) waves (e.g., below 1 GHz) requires a large material size or thickness. Currently, with the development of microwave communication and detection technology, P band microwaves, ranging from 300 to 1000 MHz, have been widely used in wireless communication and defense radar systems because of their potentials for wide coverage and strong wave penetration. As the Internet of Things (IoT) applications gradually emerge, sub-GHz frequency wireless communication occupies a dominant position. Hence, the EM interference or EM compatibility issues and radiation protections for the P band microwaves are of great importance. On the other hand, for defense applications, radars working in the P band have become a research focus, because the P band microwaves have an anti-stealth advantage [10], resulting in an urgent demand for high-efficiency EM absorbers working in the P band.

Due to the increased wavelength, a large size or thickness accompanied with heavy weight is required for conventional absorption materials to achieve EM energy absorption in the P band, which limits their practical applications. Metamaterial absorbers (MMAs) composed of subwavelength resonant unit cells have attracted extensive attention due to their thin thickness, controllable and scalable working frequencies, as well as excellent absorption performance [11,12,13,14,15,16]. For examples, Yoo et al. [17] proposed a flexible and elastic MMA supporting an absorption peak at 400 MHz in the P band. Khuyen et al. [18] presented an ultra-subwavelength thickness MMA achieving three different absorption peaks at very low frequencies. However, the narrow absorption band for the designed absorbers will prevent their wide practical applications.

Rozanov analyzed the relationship between the thickness of the metal-backed absorber and its absorption bandwidth [19], and he pointed out that magnetic substrates can provide a broadband absorber with a smaller thickness than dielectric substrates. Thus, in some studies, absorbers have been designed based on the combination of magnetic materials and metallic resonant structures (e.g., the frequency selective surface (FSS), an artificially designed structure working in a certain band) to reduce the overall thickness while maintaining broadband absorption performance [20,21,22]. However, they still could not operate for the lower microwave frequency band, especially for the P band. By introducing tunable components, such as varactors [23,24,25,26] and PIN diodes [27,28], into the metamaterial absorbers design, they can exhibit tunable absorption bandwidth upon external stimulants of bias voltages. As the bias voltages change, the absorber impedance matches the free space at different frequencies, leading to high-efficiency absorption covering an effective broad working bandwidth that may be used for lower microwave applications. For example, Zhang et al. [29] proposed an ultrabroadband double-side and dual-tuned absorber for ultrahigh-frequency (UHF) bands with a total thickness of about 25 mm. However, it remains still challenging to further reduce the absorber thickness and practically realize high-performance absorption in much lower frequencies.

Here, we propose an ultrathin tunable metamaterial absorber based on the co-design of magnetic nanomaterials, metallic structure resonances, and active components (i.e., varactors with tunable capacitances), which could be potentially developed as self-adaptive or intelligent absorbers with smart high-speed tunability when it is integrated with the detection system. The utilization of magnetic nanomaterials can significantly reduce the overall thickness of the absorber, which is combined with the varactors to enable an active control of the wave absorption. As a result, a continuously adjustable absorption above 90% can be achieved from 0.41 to 1.02 GHz within a total thickness of only 5 mm, or 1/146 of the lowest operating wavelength. Both equivalent circuit method (ECM) and full-wave simulations are performed to analyze the working mechanism. Experiments are carried out to verify the excellent absorption performance of the metamaterial absorber, and agreements are observed between theoretical, simulated, and measured results. The proposed general design method could be utilized for the co-design of versatile resonant structures and other magnetic materials, which may find applications such as scattering reduction, EMC, etc.

## 2. Design and Simulation

### 2.1. EM Properties of Magnetic Nanomaterial

**Figure 1:**
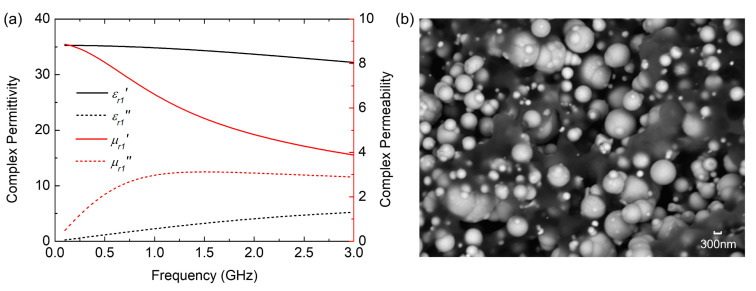
(**a**) The EM parameters of the magnetic nanomaterial. (**b**) The SEM image of the magnetic nanomaterial.

Magnetic materials can effectively extend the absorption bandwidth within a small thickness, because their increased permeability not only improves the possibility of achieving ultrawideband impedance matching to free space but also provides extra magnetic losses compared with dielectric materials. Making full use of high microwave permeability, high magnetic losses, and an appropriate ratio between the permeability and permittivity, the magnetic material can achieve good absorption performance [30]. Considering these, the customized magnetic nanomaterial, a mixture of carbonyl iron powders and resins, is employed for designing the metamaterial absorber. The relative complex permeability ($\mu_{r1}=\mu_{r1}^{\prime }-j\mu_{r1}^{\prime \prime }$) and permittivity ($\varepsilon_{r1}=\varepsilon_{r1}^{\prime }-j\varepsilon_{r1}^{\prime \prime }$) of the magnetic nanomaterial are shown in Figure 1a, which is obtained by using the coaxial-line method and a vector network analyzer (Agilent N5244). The scanning electron microscopy (SEM) image of the magnetic nanomaterials is shown in Figure 1b, and it is obvious that carbonyl iron powders on the nanometer scale are uniformly distributed in the resin, which provides homogeneous and stable material properties. Sufficient carbonyl iron powders mixed in the resin make the magnetic material possess high real and imaginary parts of the permeability, which will produce large magnetic losses when interacting with the EM wave. As depicted in Figure 1, the measured results show that the magnetic nanomaterial characterizes the frequency-dependent high permeability of both real and imaginary parts, indicating its potential for the high absorption design [31].

### 2.2. Element Design

**Figure 2:**
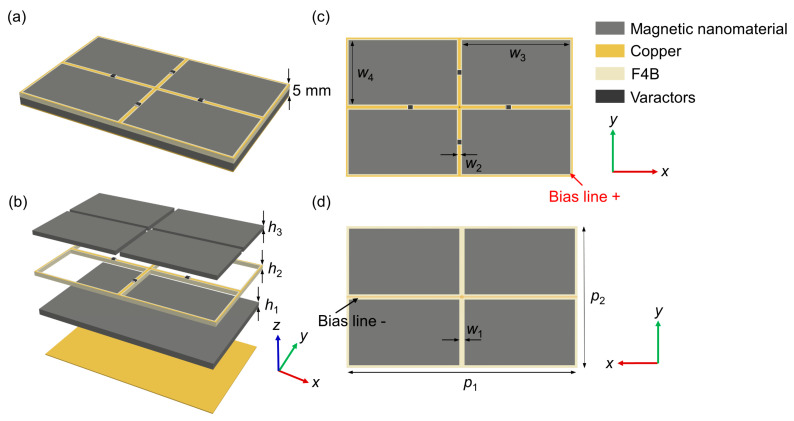
(**a**) Perspective view of the designed unit cell with a total thickness of 5 mm. (**b**) The construction of the unit cell of the proposed metamaterial absorber. (**c**) Top view of the upper layer. (**d**) Back view of the upper layer.

The proposed absorber is a combination of magnetic nanomaterials and tunable resonant structures, which is backed by a metallic plate to totally block the wave transmission. As Figure 2a plots, the metallic structure shaped into periodic frame structures is embedded in magnetic materials, and the magnetic material is divided into four cuboids, with the thickness of *h*_3_. The periodic lengths of the unit cell in the *x*- and *y*-direction are *p*_1_ and *p*_2_, respectively. Figure 2a plots the perspective view of the designed absorber unit cell. The frame structure with a thickness of *h*_2_ is placed on the bottom magnetic material layer with the thickness of *h*_1_, and the dielectric layer (F4B) supporting metallic pattern is chosen with a dielectric constant of 2.65 and a loss tangent of 0.001. The metallic structures attached to the substrate are made of copper film with a conductivity of 5.8 × 10^7^ S/m and thickness of 0.018 mm. Four varactors (SMV2020-079LF) whose capacitance can vary from 3.2 to 0.35 pF under the input bias voltage of 0–20 V are used to connect the split gap of the metal strips along the *x*- and *y*-direction, as shown in Figure 2c. The widths of the substrate frame and the metal strips are *w*_1_ and *w*_2_, respectively. The center of the metal strips on the upper side of the dielectric substrate is connected to the bias line on the other side of the substrate by a metallic via hole, as illustrated in Figure 2d. Commercial software of CST Microwave Studio is employed to perform numerical simulations of the unit cell structure. In the full-wave simulations, Floquet boundary conditions are applied to the *x*- and *y*-direction, while an open (add space) boundary is applied to the *z*-direction, with the incidence propagating along the *z*-direction. The mesh size is set to be automatically adjusted and iterated with a convergence threshold value of 0.01 (linear), which refers to the maximum deviation between the last two curves calculated by the solver. Via careful optimization, the final parameter values of the designed absorber are listed in Table 1. The total thickness of the absorber is 5 mm: approximately a 1/146 wavelength compared to the lowest working frequency. In the simulation, the absorbance of the absorber can be calculated as *A*(ω) = 1 − *T*(ω) − *R*(ω), where the transmittance *T*(ω) = |*S*_21_|^2^ and reflectance *R*(ω) = |*S*_11_|^2^ are obtained from the frequency-dependent complex S-parameters. The transmittance *T*(ω) can be suppressed to zero due to the existence of metallic ground. Hence, the absorption can be simplified to *A*(ω) = 1 − *R*(ω), which indicates that the absorption performance is only determined by the reflectivity.

**Figure 3:**
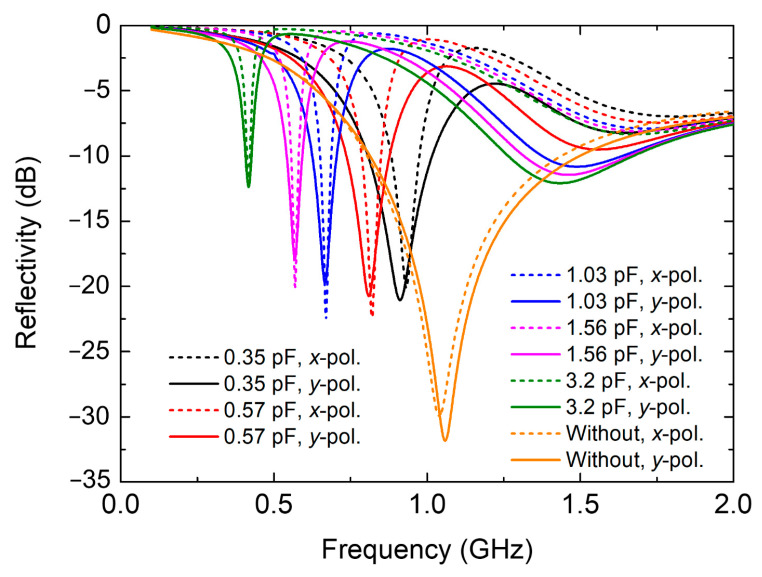
Simulated reflectivity of the proposed absorber. The varactors are set with different capacitances under *x*- and *y*-polarization normal incidences. The lines denoted by “without” represents that the structure is only composed of magnetic materials without loading frame-shaped structures.

The simulated reflectivity of the absorber with various capacitances is compared with that of the magnetic absorber without metallic structures, and the results are plotted in Figure 3. Clearly, the absorber composed of bare magnetic materials can only realize an absorption peak above 1 GHz. After introducing the frame-shaped structure, the resonant peak shifts toward lower frequencies of the P band. In particular, as the capacitance of varactors increases, the absorption frequency shows a redshift, but the resonant depth and bandwidth decrease. Compared with results for *y*-polarization (solid line) normal incidence, the absorption bandwidth under *x*-polarization (dotted line) is slightly reduced, because the bias lines for inputting external voltages are designed along the *x*-direction (Figure 2d) that have an influence on the absorption performance. Despite this, the center operating frequencies are nearly the same for both polarizations that can be continuously tuned from 0.41 to 1.02 GHz with −10 dB reflectivity, approximately approaching 85.3% fractional bandwidth.

### 2.3. Analysis of the Working Mechanism

**Figure 4:**
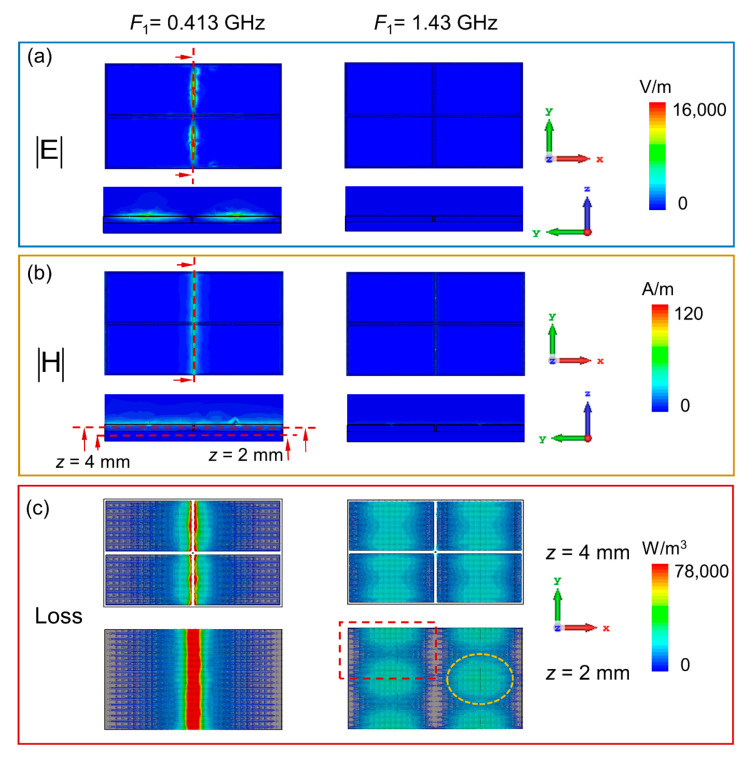
Distributions of (**a**) electric field, (**b**) magnetic field, and (**c**) power loss under the *y*-polarization normal incidence at 0.413 GHz and 1.43 GHz. The cross-section is cut along the red dotted line.

To further investigate the energy dissipation mechanism of the absorber, we take the case of the structure with the varactor capacitance of 3.2 pF under the *y*-polarization incidence as an example to analyze the EM fields and power loss distributions at the two resonant frequencies. The first column of Figure 4 shows the electric field, magnetic field, and power loss distributions at 0.413 GHz, which is the first resonance observed from the reflectivity curve. At this frequency, the metallic strip structures along the *y*-direction resonate with the incident wave, and EM fields are concentrated in the place where the varactors are loaded, as illustrated in Figure 4a,b. Figure 4c shows that the power loss distributions are concentrated in the magnetic materials around the resonance of the metallic frame structures. The frame structure is embedded in the magnetic cuboids so that the excited EM field can be better dissipated through magnetic losses, leading to a high absorption in P band. Moreover, the absorption frequency is determined by the varactor capacitance, and then, we can adjust the absorption frequency band to fit different working requirements by electrically tuning the voltage applied onto the structure. The second absorption frequency of the reflectivity curve is 1.43 GHz, and the EM fields and power loss distributions are plotted in the second column of Figure 4. There is no obvious concentration of EM fields, and the power loss is distributed at the junction between magnetic material stepped structures along the polarization direction, as shown by the yellow dotted circle in Figure 4c. The magnetic material layer of the unit cell can be divided into four stepped structure components, which are marked with a red dotted rectangle in Figure 4c. The gap between magnetic material elements leads to the coupling and loss of incident electromagnetic waves, resulting in an absorption at this frequency. According to the above analysis of the field and power loss distributions, it is obvious that the active frame-shaped structure and magnetic material components work together to achieve P band absorption, while the second absorbing frequency is mainly attributed to the loss in magnetic materials.

**Figure 5:**
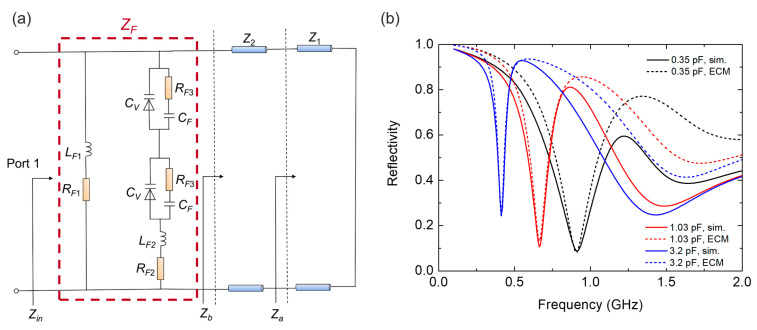
(**a**) The equivalent circuit model of the absorber. (**b**) Comparison of the reflection coefficient between the CST simulation and ECM calculation.

The ECM can be used to provide clear insight into the working mechanism from a circuit point of view. Different to the above field analysis that focuses the origins of the structural resonant behaviors, ECM provides analytical predictions of the absorption performance. Previous ECM analysis is based on bare dielectric substrates without magnetic materials. For the lossy substrate with complex EM parameters (i.e., the magnetic material in this study), the equivalent circuit model will be more intricate, and additional loss induced by magnetic materials should be considered in a dispersive form because the EM parameters of magnetic materials are frequency-dependent. The general equivalent circuit model of the proposed absorber is established as shown in Figure 5a, which consists of transmission lines and RLC components. The bottom magnetic nanomaterial layer and the second layer composed of the combination of F4B dielectric substrates and magnetic materials can be treated as fractions of the transmission line with certain characteristic impedances *Z*_1_ and *Z*_2_, respectively. The surface metallic strips loaded with varactors are modeled as a series-connected RLC circuit, as shown in the red dashed box in Figure 5a. Since a ground metallic plate is used as the bottom layer, the equivalent circuit model is terminated by a short load.

According to transmission line theory, when looking from port ‘*a*’ (2 mm apart from the short load) toward the bottom ground plane, the input impedance *Z_a_* can be derived as:

(1) $$Z_{a}=j\sqrt{\frac{\mu_{r1}}{\varepsilon_{r1}}}Z_{0}\tan(k_{1}h_{1}),$$

where Z_0_ = 377 Ω represents the characteristic impedance of free space and $k_{1}=\omega \sqrt{\varepsilon_{0}\mu_{0}\varepsilon_{r1}\mu_{r1}}$ is the wavenumber in the medium. *ε_r_*_1_ and *μ_r_*_1_ are the relative permittivity and permeability of the magnetic material, respectively, which vary with the frequency, as shown in Figure 1. Similarly, we can obtain the effective impedance when looking at port ‘*b*’ toward port ‘*a*’, and the input impedance *Z_b_* is:

(2) $$Z_{b}=\sqrt{\frac{\mu_{r2}}{\varepsilon_{r2}}}Z_{0}\times \frac{Z_{a}+j\sqrt{\frac{\mu_{r2}}{\varepsilon_{r2}}}Z_{0}\tan(k_{2}h_{2})}{\sqrt{\frac{\mu_{r2}}{\varepsilon_{r2}}}Z_{0}+jZ_{a}\tan(k_{2}h_{2})},$$

where *ε_r_*_2_ and *μ_r_*_2_ are the equivalent permittivity and permeability of the second layer, and $k_{2}=\omega \sqrt{\mu_{0}\varepsilon_{0}\mu_{r2}\varepsilon_{r2}}$ is the wavenumber in this medium. The equivalent EM parameters are calculated by the following equations:

(3) $$\varepsilon_{r2}=\varepsilon_{r2}^{\prime }-j\varepsilon_{r2}^{\prime \prime },\;\mu_{r2}=\mu_{r2}^{\prime }-j\mu_{r2}^{\prime \prime },$$

(4) $$\varepsilon_{r2}^{\prime }=m_{1}\varepsilon_{r1}^{\prime }+(1-m_{1})\varepsilon_{e}^{\prime },\;\varepsilon_{r2}^{\prime \prime }=m_{1}\varepsilon_{r1}^{\prime \prime }+(1-m_{1})\varepsilon_{e}^{\prime \prime },$$

(5) $$\mu_{r2}^{\prime }=m_{1}\mu_{r1}^{\prime }+(1-m_{1})\mu_{e},\;\mu_{r2}^{\prime \prime }=m_{1}\mu_{r1}^{\prime \prime },$$

where *m*_1_ is the volume ratio of the magnetic materials to the total second layer of the composite substrate. Additionally, $\varepsilon_{e}^{\prime }=2.65$, $\varepsilon_{e}^{\prime \prime }=0.001$, and *μ_e_* = 1 are the EM parameters of the F4B dielectric layer used for supporting the metallic frame structure.

For the surface structures of the top layer, *L_F_*_1_ and *R_F_*_1_ are equivalent circuit parameters of metal strips, while *L_F_*_2_ and *R_F_*_2_ are equivalent circuit parameters of metal strips with the split. The split on the metal strips is modeled as *C_F_* and *R_F_*_3_ in parallel connection to the varying capacitance *C_v_* of varactor. The simplified model of the varactor *C_v_* is shown in Figure 5b, where *R* = 2.5 Ω, *L* = 0.7 nH, and *C* varies from 0.35 to 3.2 pF according to the data sheet. Different from the traditional equivalent circuit model analysis of metallic structures with negligible loss, here, additional complex resistors *R_F_*_1_ and *R_F_*_2_ should be added to the circuit model due to the loss of magnetic substrates. Because the equivalent permittivity (*ε_r_*_2_) and permeability (*μ_r_*_2_) of the composite substrate under the surface structures change with the frequency and the current excited by the incident wave on the metallic structures will be coupled and interact with the composite substrate, the equivalent RLC parameters operate in a frequency-dependent manner that acts as a function of the EM parameters of the magnetic substrate [32]:

(6) $$L_{F1}=A_{1}\mu_{0}(\mu_{r2}^{\prime }+1)\;nH,\;R_{F1}=A_{2}\mu_{0}\mu_{r2}^{\prime \prime }f\;\Omega ,$$

(7) $$L_{F2}=A_{3}\mu_{0}(\mu_{r2}^{\prime }+1)\;nH,R_{F2}=A_{4}\mu_{0}\mu_{r2}^{\prime \prime }f\;\Omega ,$$

(8) $$C_{F}=A_{5}\varepsilon_{0}\frac{{\left(\varepsilon_{r2}^{\prime }+1\right)}^{2}+\varepsilon_{r2}^{\prime \prime }{}^{2}}{\varepsilon_{r2}^{\prime }+1}\;pF,\;R_{F3}=\frac{A_{6}}{\varepsilon_{0}}\frac{\varepsilon_{r2}^{\prime \prime }}{({\left(\varepsilon_{r2}^{\prime }+1\right)}^{2}+\varepsilon_{r2}^{\prime \prime }{}^{2})f}\;\Omega .$$

The coefficients A_1_ to A_6_ are constant values determined by the overall absorber structure. During the modeling process, they are optimized by fitting the simulated reflection curves through a genetic algorithm, and the optimized values are: A_1_ = 4.98 × 10^6^, A_2_ = 1.042 × 10^−2^, A_3_ = 5.03 × 10^6^, A_4_ = 1.011 × 10^−2^, A_5_ = 1.244 × 10^9^, A_6_ = 99.91.

Considering the surface structures of the proposed absorber as an effective impedance *Z_F_*, the total input impedance of the absorber *Z_in_* can be written as

(9) $$Z_{in}=\frac{Z_{F}Z_{b}}{Z_{F}+Z_{b}}.$$

Then, the reflection coefficient is calculated by

(10) $$R=\left|\frac{Z_{in}-Z_{0}}{Z_{in}+Z_{0}}\right|.$$

The above equations reveal the dependence of the reflection coefficient on the structural geometric parameters and EM properties of the substrate materials. The equivalent circuit model validates that the reflectivity curve of the first resonant peak calculated by the ECM is consistent with the CST simulation, as shown in Figure 5b. The second absorption peak, however, is mainly attributed to interaction between magnetic nanomaterial elements, as discussed in the field analysis; thus, ECM cannot precisely calculate its absorption performance. This explains why the reflection curves do not agree well at the second absorption peak around 1.5 GHz.

### 2.4. Angular Performance

**Figure 6:**
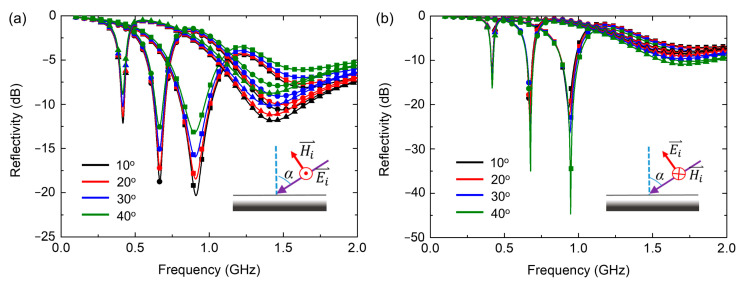
Simulated reflectivity of the absorber under *y*-polarization oblique incidence for (**a**) the TE mode and (**b**) the TM mode. The square, circle and up-triangle symbols represent that the varactor capacitance is 0.35 pF, 1.03 pF and 3.2 pF, respectively. Insets show the definition of TE and TM mode.

As angular sensitivity is essential for practical applications of microwave absorbers, we also investigate the reflection performance of the absorber under different oblique incident angles with various varactor capacitances, as shown in Figure 6. Both transverse electric (TE) and transverse magnetic (TM) polarized incidences are studied. For the TE mode incidence, the electric vector is perpendicular to the incident plane, while the magnetic vector is perpendicular to the incident plane for the TM mode. We can observe from Figure 6a that for TE polarized incidence, the absorption performance decreases with the increase in the incident angle, but it can still achieve reflectivity nearly below −10 dB until the incident angle up to 40° for all the tunable frequencies. Meanwhile, for the TM mode case, the absorbing efficiency is enhanced when increasing the incident angle. Such a difference between TE and TM polarized incidences is due to that when the incident angle increases, the electric and magnetic vectors of the TE polarized incidence change quite differently from those of the TM polarized incidence. For the TE wave, the magnetic field will gradually rotate out of the *xoy* plane as the incident angle increases; thus, the effective magnetic response of the unit cell is reduced, which will decrease the absorbing performance of the structure because the absorber mainly relies on magnetic losses. However, the incident magnetic field can remain parallel to the *xoy* plane and achieve in-plane interaction with the structure, while the electric field gradually rotates out of the plane as the incident angle increases for the TM mode. The effective electric response of the unit cell is accordingly reduced, which contributes to the enhancement of absorption for oblique TM waves [33]. The reflectivity curve shows good angular stability in the range of 0° to 40° for both TE and TM polarization, indicating the good angular performance of the metamaterial absorber.

### 2.5. Performance Evaluation

**Figure 7:**
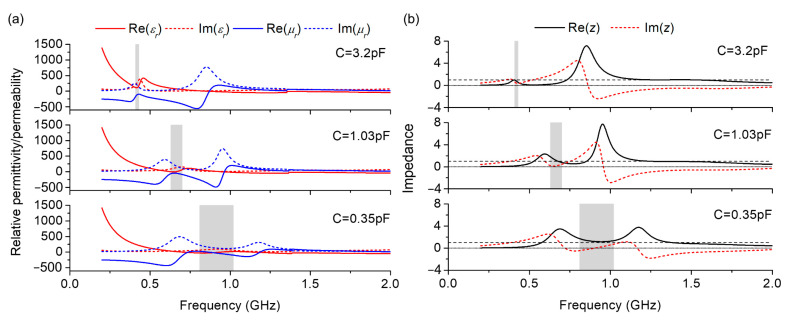
Retrieved (**a**) constitutive parameters and (**b**) effective wave impedance for different loaded capacitors of the absorber.

The proposed metamaterial absorber can be treated as an effective medium, and its effective EM parameters can be retrieved from simulated reflection (*S*_11_) and transmission (*S*_21_) data via a homogenization algorithm [34,35]. Because the absorber is backed by the ground metallic plate, to obtain the transmission results, here, four small holes (radius of 1 mm) are opened at the corners of the ground metallic plate to allow small transmission as a perturbation. Although the transmission is very small, its phase and amplitude responses are sufficient for the parameter extraction. The retrieval formulas are as follows [36]:

(11) $$n=\frac{1}{kd}\cos^{-1}\left[\frac{1}{2S_{21}}(1-S_{11}^{2}+S_{21}^{2})\right],$$

(12) $$z=\sqrt{\frac{{\left(1+S_{21}\right)}^{2}-S_{21}^{2}}{{\left(1-S_{21}\right)}^{2}-S_{21}^{2}}},$$

(13) $$\varepsilon_{r}=n/z,\;\mu_{r}=nz,$$

where *n* and *z* are the effective refraction index and impedance, respectively, and *d* is the total thickness of the structure. Then, the retrieved constitutive parameters and effective wave impedance of the designed absorber under the *y*-polarized normal incidence with different loaded capacitors are shown in Figure 7, and the gray region represents the absorption frequency band (below −10 dB).

Based on the effective medium description, theoretical requirements should be satisfied by the effective parameters for an electromagnetic absorber to achieve near-perfect absorption [37]:

(14) {Re(εr)=Re(μr)Im(εr)=Im(μr)≫1kd.

This indicates that lower-frequency absorption is more difficult when simultaneously requiring a thicker thickness or larger imaginary parts of the effective parameters, because the wave vector *k* is proportional to the frequency. In addition, it should be noted that Equation (14) cannot be strictly satisfied at all frequencies, owing to the inherent constraints set by the Kramer–Kronig relation [38], which links the real and imaginary parts of the effective permittivity and permeability over the whole frequency range. For the proposed absorber, as shown in Figure 7a, the imaginary parts of the effective EM parameters are large due to the magnetic nanomaterial, and the real (solid line) and imaginary (dotted line) parts of *ε_r_* and *μ_r_* are equal, respectively, in different frequency bands (gray region) with different loaded capacitances; thus, the absorption resonances are changed by varying the varactor capacitance to meet the requirements of near-perfect absorption at different frequencies. Therefore, compared with nonmagnetic or passive absorbers, the designed absorber can realize broadband continuous tunable wave absorption at low frequencies with a much thinner thickness.

For a physically realizable broadband absorber, the Rozanov limit indicates that for any absorber under the normal incidence, its total thickness *d* must be larger than a theoretical limit for the given frequency response of the absorption [19], i.e.,

(15) $$d\geq \frac{1}{2\pi^{2}\mu_{s}}\left|\int_{0}^{\infty }\ln\left|\rho (\lambda )\right|d\lambda \right|>\frac{1}{2\pi^{2}\mu_{s}}\left|\ln(\rho_{0})\right|(\lambda_{\max}-\lambda_{\min}),$$

where *ρ* is the reflection coefficient, *λ* is the free space wavelength, and *μ_s_* = Re(*μ_r_*)|*_λ_*_→∞_ is the static permeability of the structure. For a nonmagnetic (*μ_s_* = 1) and non-tunable absorber, the theoretical limit is estimated to be 25.5 mm to achieve absorption below −10 dB from 0.41 to 1.02 GHz.

As the response of the material always lags behind the incident wave (i.e., “cause” leads to “result”), the absorption performance of the material is limited by the “causality limit”. The causality-dictated minimum thickness [37] is given by

(16) $$d_{\min}=\frac{1}{2\pi^{2}\mu_{s}}\left|\int_{0}^{\infty }\ln\left|\rho (\lambda )\right|d\lambda \right|.$$

**Figure 8:**
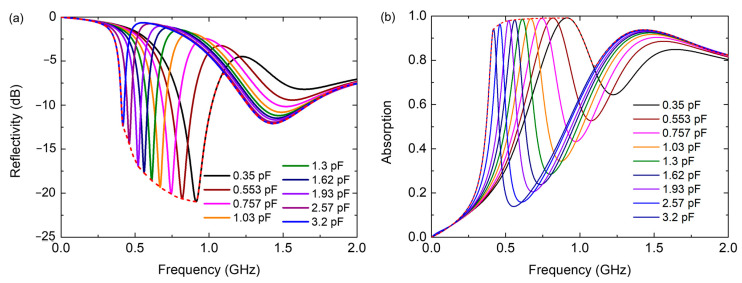
The simulated (**a**) reflection and (**b**) absorption curves by tuning the varactor capacitance. Dotted line shows the envelope of the achievable absorption region.

Here, the parameter *μ_s_* is set as 8.9 as observed from Figure 1. By substituting the simulated reflection spectrum of the structure with different loaded capacitances into Equation (16), i.e., the results of C_m_ = 0.35 pF, C_m_ = 1.03 pF, and C_m_ = 3.2 pF shown in Figure 8a, we can obtain the theoretical minimum thickness of *d*_min_ = 4.06 mm, *d*_min_ = 4.11 mm and *d*_min_ = 3.93 mm, which is dictated by the causality limit to realize the required reflection properties of each curve in Figure 8a, respectively. The actual thickness *d* = 5 mm of our absorber is slightly larger than the theoretical minimum thickness.

The proposed metamaterial absorber possesses real-time tunable absorption responses that are controlled by the bias voltage to enable high-efficiency absorption at different low frequencies. Hence, it could be used adaptively for different requirements of the absorption band, and the effective working bandwidth could be evaluated by the achievable absorption range under different varactor capacitances. The envelope of the reflection curve within the adjustable range is shown by the dotted red curve in Figure 8a, while the result of absorption is shown in Figure 8b. The tunable absorber can achieve accessible absorption performance through dynamic control of the bias voltage. By substituting the envelope curve of the reflection spectrum into Equation (16), we can obtain the theoretical minimum thickness *d*_min_ = 8.12 mm. Although the metamaterial absorber cannot simultaneously realize high-efficiency absorption in all frequencies within the achievable range, it can operate in a time-multiplexing manner that could still be used in many applications.

## 3. Experiment Results and Discussion

**Figure 9:**
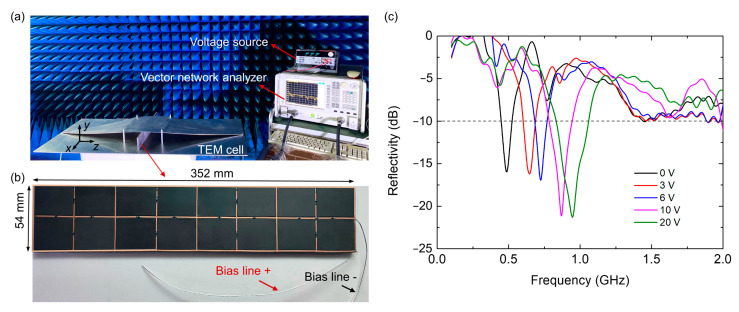
(**a**) Photograph of the measurement setup. (**b**) The photograph of the fabricated prototype. (**c**) Measured reflectivity of the fabricated prototype under *y*-polarization normal incidence.

To verify the design principle and the simulation results, we fabricated the absorber sample and tested it in the customized TEM cell, which is a waveguide structure that supports the propagation of the quasi-TEM mode along the *z* direction. Such a measurement set-up offers an efficient way to characterize the EM performances of periodic structures [39,40,41] just with a small-sized sample especially at the lower microwave band, which is an alternative to the free-space measurement in large microwave chamber. In the experiments, the frequency responses are transformed to the time domain, and a time gating is employed to reduce the multiple reflection effects for the improvement of measurement accuracy [42]. Figure 9a illustrates a photograph of the measurement setup and the fabricated prototype. The fabricated sample consists of 1 × 4 unit cells with a size of 54 × 352 mm^2^, as shown in Figure 9b.

The measured reflectivity results are shown in Figure 9c. The designed absorber can achieve continuously tunable absorption from 0.45 to 1.06 GHz under the *y*-polarization normal incidence when the input bias voltage is changed from 0 to 20 V. Compared with the simulated results, there are slight frequency shifts in the measurement, which is probably caused by the inaccuracy of sample fabrication, imperfection of the measurements, value variations, and parasitic effects of the lumped elements. The reflection curve slightly above 0 dB at much low frequencies is mainly due to the calibration deviation. Considering these influences and tolerances, the measured result roughly agrees with the simulated result, demonstrating the capability of the fabricated sample to efficiently achieve tunable absorption in the P band. Although the absorber we designed possesses excellent performance for absorbing applications below 1 GHz, some scenarios may need to operate for frequencies larger than 1 GHz. To circumvent this limitation, we can increase the number of layers and thickness of magnetic material structures so as to expand the working bandwidth above 1 GHz. However, the magnetic materials may not be used for very high frequencies, as the magnetic loss (or the imaginary part of the permeability) decreases as the frequency increases.

## 4. Conclusions

We present a reconfigurable absorber combining magnetic nanomaterials, resonant structures, and active components of varactors, which can realize continuously and electrically tunable absorption in the lower microwave band from 0.41 to 1.02 GHz by adjusting the input bias voltage. The most advantage of the absorber is its ultrathin thickness and broadband absorption at lower frequencies. EM field analysis and ECM analysis are applied to reveal the underlying working mechanism of the metamaterial absorber, and good angular stability is achieved for the oblique incidence. Experiments are conducted to validate the design principle, and consistencies are observed between simulated and measured results. The low-frequency microwaves, especially below 1 GHz, have been widely used in wireless communication and defense radar systems, resulting in an urgent demand for high-efficiency EM absorbers working at this frequency band for the EMC or stealth technique. The proposed design method and metamaterial absorber have a thin–thickness working for low frequencies, which may open up a plethora of interesting applications, such as anti-radar environment, radio-frequency identification (RFID) systems, sub-GHz wireless systems and so on.

## Figures and Tables

**Table 1:** Parameters of the proposed MMA.

Parameters	*h*_1_	*h*_2_	*h*_3_	*p*_1_	*p*_2_
**Value (mm)**	3	2	2	88	54
**Parameters**	*w*_1_	*w*_2_	*w*_3_	*w*_4_	Total thickness
**Value (mm)**	2	1	42	25	5

## Data Availability

The data that support the findings of this study are available from the corresponding authors upon reasonable request.
